# Relationship between Maternal Immunological Response during Pregnancy and Onset of Preeclampsia

**DOI:** 10.1155/2014/210241

**Published:** 2014-06-02

**Authors:** Alicia Martínez-Varea, Begoña Pellicer, Alfredo Perales-Marín, Antonio Pellicer

**Affiliations:** ^1^Department of Obstetrics and Gynecology, Hospital Universitario y Politécnico La Fe, Avenida Bulevar Sur s/n, Valencia 46026, Spain; ^2^Department of Obstetrics and Gynecology, Hospital General Universitario, Avenida Tres Cruces 2, Valencia 46014, Spain

## Abstract

Maternofetal immune tolerance is essential to maintain pregnancy. The maternal immunological tolerance to the semiallogeneic fetus becomes greater in egg donation pregnancies with unrelated donors as the complete fetal genome is allogeneic to the mother. Instead of being rejected, the allogeneic fetus is tolerated by the pregnant woman in egg donation pregnancies. It has been reported that maternal morbidity during egg donation pregnancies is higher as compared with spontaneous or *in vitro* fertilization pregnancies. Particularly, egg donation pregnancies are associated with a higher incidence of pregnancy-induced hypertension and placental pathology. Preeclampsia, a pregnancy-specific disease characterized by the development of both hypertension and proteinuria, remains the leading cause of maternal and perinatal mortality and morbidity. The aim of this review is to characterize and relate the maternofetal immunological tolerance phenomenon during pregnancies with a semiallogenic fetus, which are the spontaneously conceived pregnancies and *in vitro* fertilization pregnancies, and those with an allogeneic fetus or egg donation pregnancies. Maternofetal immune tolerance in uncomplicated pregnancies and pathological pregnancies, such as those with preeclampsia, has also been assessed. Moreover, whether an inadequate maternal immunological response to the allogenic fetus could lead to a higher prevalence of preeclampsia in egg donation pregnancies has been addressed.

## 1. Introduction

Maternal immunological response during pregnancy is essential to maintain this state. It implies tolerance to the semiallogeneic fetus, which possesses half maternal genes and half paternal genes. The more genetically distinct the fetus is, the greater the immunological tolerance becomes during pregnancy. This occurs during pregnancies by egg donation (ED), when the fetus is allogenic. Such tolerance also intervenes in modulating pregnancy-related pathologies, including preeclampsia [1]. As it complicates up to 8% of pregnancies, preeclampsia is the leading cause of maternal and perinatal mortality and morbidity. Adverse perinatal outcomes, such as prematurity and intrauterine growth restriction, are related to this condition. Preeclampsia is a pregnancy-specific disease characterized by the development of both hypertension and proteinuria. Occasionally, the disease progresses into a multiorgan cluster of varying clinical features. Predisposing disorders include chronic hypertension, diabetes, and obesity. Moreover, African-American and Filipino women and a low socioeconomic status are associated with increased risk. Although the precise etiology of the disorder is still unknown, deficient early placentation is particularly associated with early onset preeclampsia [2]. In fact, abnormal placentation is thought to be immunologically mediated [2, 3]. As prevention and prediction of preeclampsia are still not possible, symptomatic clinical management should focus on preventing maternal morbidity (e.g., generalised seizures of eclampsia) and mortality [2].

From the epidemiological viewpoint, there is a higher incidence of pregnancy-induced hypertension in pregnancy by ED, which oscillates between 16% and 40% of all cases [4]. Compared to pregnancy by autologous* in vitro* fertilization (IVF), the prevalence of hypertensive complications is 26–37% as opposed to 8%, with an odds ratio (OR) of 7.1 for the ED group [4]. Furthermore, the incidence of pregnancy-induced hypertension is higher in ED-pregnant women, who are not related to the donor and have not been previously exposed to the donor's sperm [3–5].

The aim of this review is to analyze the maternofetal immunological tolerance phenomenon and its possible relation with preeclampsia onset, because this could justify the higher prevalence of this pathology in ED pregnancies as a result of an inadequate maternal immunological response to the allogeneic fetus.

## 2. Maternofetal Immunology

Numerous fetal, maternal, and placenta-based mechanisms protect the fetus against the maternal immune system. The fetal tissue that invades maternal territory is characterized as being poorly immunogenic. The trophoblast hardly expresses the molecules of the main histocompatibility complex (MHC) or the human leukocyte antigen (HLA), which contribute variability within the same species. This and other immunoregulatory mechanisms endeavor to avoid fetal cells from being innately rejected upon their arrival [6, 7].

Even before implantation, the receptive maternal setting for the host is reflected in the uterus. Scarce cytotoxic activity to foreign agents and the outstanding capacity to segregate cytokines of uterine natural killer (uNK) cells in the maternofetal interphase intervene in the extensive hemodynamic remodeling that the pregnant uterus undergoes [6, 7]. A systemic maternal response is prepared even before the zygote reaches the uterus, which is based on the expression of the cytokine profile that is characteristic of T helper lymphocytes (Th) type 2 (Th2) [6, 7].

### 2.1. Implantation and Maternal Immunological Response on the Maternofetal Surface

During human placentation, three main changes take place in the pregnant uterus. First, the endometrium is differentiated in the dense cell matrix known as the decidua. Second, the decidua and the underlying myometrium are invaded by fetal trophoblastic cells. Third, a subtype of these cells, fetal extravillous cytotrophoblast (EVT), penetrates maternal vessels, which alters and replaces the endothelium and part of the muscle layer. In this way, the maternal uterine arteries are transformed into wide low-resistance vessels due to the destruction of their muscle layer, which leads to increased maternal flow to the placenta [8, 9].

The invasive nature of hemochorial placentation implies direct contact between maternal and fetal cells. Placental villosities, composed of the cytotrophoblast and covered by syncytiotrophoblast, are immersed in the circulating maternal blood that comes into contact with its constituent immune cells. The exact mechanisms involved in maternal immunological tolerance which allow for a semiallogeneic or allogeneic pregnancy in ED [10–12] are still unknown. Alteration due to excessive or deficient placentation may lead to a pathological pregnancy. For example, some authors postulate that the well-known maternal inflammation associated with preeclampsia may arise from a high concentration of the syncytiotrophoblast microparticles circulating in the mother's blood. These would overactivate the response of maternal monocytes through their toll-like receptors (TLR-) 1 [13–16].

In order to successfully complete implantation, the maternal decidua undergoes immunological changes. These already begin in the secretory phase of the female menstrual cycle, and they adapt to the immune response from a preconceptional stage. Among the cell components, the immunoregulatory and proangiogenic functions of uNK cells and antigen-presenter cells (macrophages and dendritic cells (DCs)) are highlighted [17].

#### 2.1.1. Uterine Natural Killer Cells

The four main populations of decidual leukocytes present in early-stage pregnancy are uNK cells, macrophages, DCs, and T-cells. Of these, the most abundant are uNK, macrophages, and T CD3+ cells (CD8+ and rarely CD4+). B-cells are virtually undetected [18].

NK cells are characterized by the expression of surface markers CD56 and CD16 and are subdivided into two populations based on the density of marker CD56 (bright-strong or dim-medium). Of the NK cells circulating in peripheral blood, 90–95% of them are highly cytotoxic and belong to the CD56 dim CD16+ phenotype. The rest of them possess CD56 bright CD16- and are highly efficient at secreting cytokines. In the decidua, the majority of NK cells possess a CD56 bright CD16- phenotype. Thus, uNK cells differ phenotypically from NK cells in peripheral blood and are characterized by poor toxicity and a good capacity to secrete cytokines and angiogenic mediators [19]. Their life cycle is limited. They rapidly proliferate during the late secretory phase of the menstrual cycle and drop in number after the halfway point of human gestation. The fact that uNK cells are present before implantation, and even in the decidua of an ectopic pregnancy, suggests that they are induced by signals regulated by stromal endocrine factors rather than by embryonic tissue [19].

uNK cells regulate trophoblast invasion through the secretion of angiogenic growth factors, cytokines, and chemokines [20, 21]. Moreover, the ability of uNK cells to kill semiallogeneic fetal cells or allogeneic cells in ED pregnancies is limited [21]. The close contact between the EVT and decidual leukocytes suggests the existence of paracrine interactions between maternal leukocytes and fetal cells [18]. Cytokines produced by uNK cells at the human fetal-maternal interface include interleukin (IL) 8, interferon-inducible-protein-10 (IP-10), and the most synthesized cytokine by uNK, regulated upon activation normal T-cell expressed and secreted (RANTES), triggers the migration of the invasive trophoblast. Angiogenic factors of uNK include vascular endothelial growth factor (VEGF) and placental growth factor (PlGF), as well as the most abundant, NKG5 [21]. The genuine interest in the role that immunity plays in vascular remodeling emerges from the study of Hanna et al. [21], who demonstrated* in vitro* and* in vivo* that uNK cells participate in uterine spiral artery remodeling by promoting angiogenesis at embryonic implantation sites by means of a gradient of cytokines and vasoactive mediators [21, 22]. Subsequent evidence has shown that during angiogenic activation, hormone factors and the hypoxic setting are also capable of regulating the production of angiogenic factors such as VEGF and their interaction with endothelial cells [23].

*Trophoblast Invasion Regulation.* Specific trophoblast recognition is carried out by uNK cells. These cells possess activator or inhibitor receptors which belong to three main families: the type-C lectin family (CD94/NKG), the killer immunoglobulin-like receptor (KIR), and immunoglobulin-like transcripts (ILT or the leukocyte immunoglobulin-like receptor) [24]. The effector functions of NK cells depend on fine tuning between these inhibitor and activator receptors, and they are considered activated when KIR receptors are constitutively expressed [19].

It has been demonstrated that extravillous trophoblast cells express maternal and paternal HLA-C. The HLA-C ligands for maternal KIR receptors are divided into two groups, C1 and C2, which are defined by a dimorphism at position 80 of the *α*1 domain. The interaction between the trophoblast HLA molecules and the KIR receptors of the uNK cells of the maternal endometrium inhibits cytotoxic activity and modulates cytokine production and growth factors by uNK cells to favor trophoblast growth, endometrium invasion, and vascular remodeling [25].

The KIR receptors family recognizes the HLA molecules of the trophoblast. So the KIR2D receptors (containing two immunoglobulin-like domains) of uNK cells are better capable of recognizing trophoblastic HLA-C than the KIR of the NK in peripheral blood. Depending on the combination of haplotypes, KIR2D can act more like an activator or more like an inhibitor [25, 26].

The genomic KIR region contains a family of highly polymorphic and homologous genes localized in chromosome 19q13.4 inside the leukocyte receptors complex. According to populational studies, the order of the KIR genes along the chromosome has mainly determined two different haplotypes: A (lacks activator receptors) and B (possesses activator and inhibitor receptors) [25, 27].

**Figure 1:**
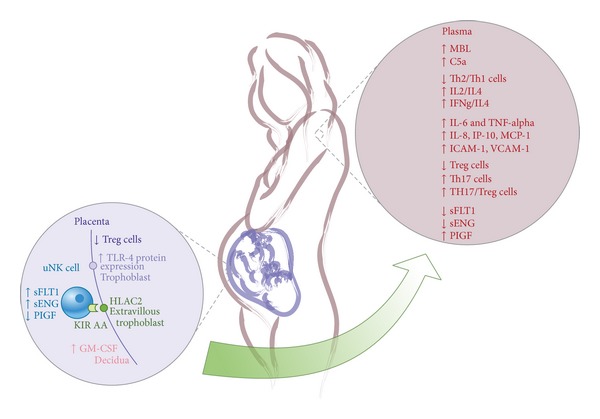
Maternofetal immune response in preeclampsia. A series of events occurs in the maternal-fetal interface in preeclampsia that result in an altered expression of different factors (PIGF, sENG, sFLT1, GM-CSF, and TLR-4) as compared to normal pregnancies. Similarly, the ratio among various populations of immune cells (Th17/Treg, Th1/Th2) differs from normality in preeclamptic patients. Regarding the complement system, preeclampsia enhances MBL and C5a synthesis. These changes are evidenced in peripheral blood in which the proinflammatory systemic environment is also seen with high IL-6 a, TNF-alpha, IL-8, IP-10, MCP-1, ICAM-1, and VCAM-1 levels. Treg: CD4+CD25+Foxp3+ T regulatory cells; TLR: toll-like receptor; HLA: the human leukocyte antigen; uNk cell: uterine natural killer cell; KIR: killer immunoglobulin-like receptor; sFLT1: soluble* fms-like* tyrosine kinase-1 factor; sENG: soluble endoglin; PIGF: placenta growth factor; GM-CSF: granulocyte-macrophage colony-stimulating factor; MBL: mannose-binding lectin; Th cell: T helper cell; IL: interleukin; IFNg: interferon gamma; TNF-alpha: tumor necrosis factor alpha; IP-10: interferon-inducible-protein-10; MCP-1: monocyte chemotactic protein-1; ICAM-1: intercellular adhesion molecule 1; VCAM-1: vascular cell adhesion protein 1; Th17: a subpopulation of TCD4+ effector cells, Thelper 17 cells.

The maternal KIR genotype can be AA (inhibitor), AB, or BB. The combination of the maternal KIR AA genotype with a fetal HLAC2 (HLAC^lys80^) increases the risk of preeclampsia [25]. As this interaction gives rise to a strong inhibitor signal, it is considered that the inhibition, and not the activation of uNK cells, predisposes to preeclampsia. uNK cells would be unable to participate in uterine arterial remodeling because they are inhibited. Therefore, the presence of activator receptors in uNK cells to protect against preeclampsia has been proposed (Figure 1) [25].

Hence uNK cells' functionality during pregnancy depends on the combination of two polymorphic genes. These are the maternal genotype for KIR (AA, AB, or BB) and fetal HLA-C haplotypes [C1 (HLAC^asn80^) or C2] [24]. During pregnancy, the frequency of the maternal KIR AA genotype increases with pathologies related to defective placentation (preeclampsia, intrauterine growth restriction, and recurrent spontaneous abortions), but only when the fetus possesses more C2 genes than the mother (e.g., maternal C1/C1 with fetal C1/C2 and maternal C1/C2 with fetal C2/C2) or when the only C2 that the fetus possesses is of paternal origin. Therefore, a deleterious effect of paternal allogeneic C2 and the early-stage role in pregnancy of these receptor/ligand pairs in reproductive failure pathogenesis have been postulated [28]. It is known that the telomeric B region of haplotype KIR B protects against these alterations in pregnancy, especially when the fetus possesses the C2 gene [27]. In general terms, different human populations present a reciprocal relation between the frequency of AA and that of HLA-C2, which suggests a selection against this combination [25].

In normal pregnancies, recognition of fetal HLA-C by receptor KIR-BB of uNK triggers the release of cytokines by uNK cells. These include transforming growth factor-beta (TGF-*β*), whose participation in immunoregulation and angiogenesis has been well-established, and angiogenic factors placenta growth factor (PIGF), and vascular endothelial growth factor (VEGF). Conversely in preeclampsia, when KIR-AA of maternal uNK cells recognize the HLA-C of the extravillous trophoblast, uNK cells display a poorer expression of these mediators [3], as well as an overexpression of antiangiogenic factors like soluble endoglin (sENG) and soluble* fms-like* tyrosine (sFLT1) kinase-1 factor. sENG inhibits TGF-*β*1 from binding to the surface of its receptors and diminishes nitric oxide-mediated endothelial signaling. sFLT1 binds to angiogenic proteins VEGF and PIGF and blocks their actions [29]. Interestingly, significantly lower PIGF levels, but with higher sFLT1 and sENG concentrations, have been demonstrated before gestation week 30 in the serum or plasma of pregnant women who have developed preeclampsia if compared with pregnant women who have not develop this disease. Therefore, they can be used as predictor markers of preeclampsia [30].

Serum levels of granulysin, a cytotoxic granule protein of NK cells and cytotoxic T lymphocytes, are significantly high in preeclamptic patients as compared with women with normal pregnancies [31, 32]. Indeed, the proportion of granulysin-producing cytotoxic T-cells notably increases in the peripheral blood of preeclamptic patients in comparison to healthy pregnant women [33]. Preeclamptic women do not show significantly different serum levels of RANTES, a cytokine produced by uNK cells at the human fetal-maternal interface, if compared with healthy pregnant women [34]. Nevertheless, the placental gene expression of RANTES has been found to be upregulated in severe early onset preeclampsia from gestational weeks 25 to 27 when compared with placental samples of uncomplicated pregnancies in similar gestational weeks [3]. Further studies are required to elucidate the exact contribution of RANTES in inducing a tolerogenic maternal immune response to allow for trophoblast survival, migration, and invasion. These studies would provide a better understanding of its role in pregnancy complications, such as recurrent spontaneous abortions or preeclampsia.

#### 2.1.2. Antigen-Presenter Cells: Dendritic Cells and Macrophages

*Dendritic Cells.* Several research lines have demonstrated the key role played by antigen-presenter cells (APC) in the maternofetal interphase during pregnancy [20].

DCs, which are the most powerful APC, are required to initiate and modulate immune responses and to induce immunological tolerance [35–37]. In humans, the density of endometrial immature DCs (CD1a+) is significantly greater than that of mature DCs (CD83+) throughout the menstrual cycle. Indeed the total number of CD1a+ DCs is much larger in the basal layer of the endometrium than in the functional layer during the secretory phase. CD1a, a highly specific and sensitive marker of immature DCs, mediates a HLA-independent antigen presentation pathway [37]. During the first trimester of pregnancy, most DCs express DC-specific adhesion receptor DC-SIGN (dendritic cell-specific ICAM-grabbing nonintegrin, classified as CD209). DC-SIGN is expressed by immature DCs in peripheral tissue [38]. In fact, the DC-SIGN expression at the maternal-fetal interface in the rhesus macaque has been reported as an early response by the primate maternal immune system to the implanting embryo [39]. Uterine DCs direct maternal receptivity by regulating decidual tissue remodeling and angiogenesis in mice. Indeed, uterine DCs play a key role in embryo implantation, when they show an immature phenotype [40].

During intrauterine and extrauterine pregnancies, the immature DC status prevails, which has been related to the interaction with uNK. The mature DC status has been associated with implantation failure [17, 38, 41], whereas the majority of decidual immature DC-SIGN+ DCs are in close contact with uNK; CD83+ mature DCs relate to CD3+ T-cells [38]. Regarding the adaptive response, DCs participate in tolerance induction as they are crucial for agonist-induced T regulatory cells (Treg) differentiation [42].

Heme oxygenase-1 (HO-1) is a microsomal enzyme with anti-inflammatory, antiapoptotic, and antiproliferative properties. It allows acceptance of allografts in mouse and its downregulation entails acute rejection. Its high expression by trophoblast cells in early pregnancy stages is well known. HO-1 reduction is related to murine pregnancy complications, such as abortion [43]. In murine pregnancies, HO-1 plays a key role in maintaining maternal DCs in an immature state. Tolerogenic immature DCs contribute to the expansion of peripheral Treg cells. Blockage of HO-1 renders DCs a mature state, which promotes the action of effector T-cells [43]. Indeed both HO-1 and its metabolite carbon monoxide promote implantation and placentation [44, 45]. HO-1 blockage leads to increased blood pressure in pregnant rats [46]. Pregnancy disorders such as preeclampsia and intrauterine growth restriction are associated with HO-1 lessening and the impaired remodeling of maternal spiral arteries [44]. Interestingly, carbon monoxide induces the proliferation of uNK cells and the remodeling of spiral arteries in pregnant hypertensive HO-1 mutant mice [44]. Accordingly in a clinical mice model of intrauterine growth restriction, carbon monoxide prevented fetal death by reducing free haem levels in circulation [45].

*Macrophages.* DCs and macrophages, present in the human endometrium, play a role in decidualization and implantation [47]. Macrophages also contribute to local immune tolerance [47–49]. After uNK, macrophages are the second most abundant population in the maternofetal interphase in both implantation and early pregnancy development [48, 49]. Macrophages congregate around spiral arteries, while the placenta develops and supports vascular remodeling by releasing proangiogenic factors, such as VEGF and MMPs and by removing apoptotic cells [50]. They also generate a wide range of cytokines, mainly for their function as APC, and they specifically produce high levels of IL-10, a well-known anti-inflammatory mediator [48]. Decidual macrophages are potential regulators of T-cell activation and activity. Hence they inhibit T-cell responses through E2 prostaglandin production, and they also produce tryptophan metabolites that can abolish T-cell proliferation [48].

Functional macrophage maturation leads to a macrophage effector phenotype, either M1 or M2 [18, 50]. M1 macrophages are activated under the influence of proinflammatory cytokines and lipopolysaccharide. In contrast, M2 macrophages are polarized by being exposed to an environment containing the cytokines of Th2 (IL-4, IL-10) and glucocorticoids [18]. M1-type macrophages, or classically activated monocytes, participate in the progression of inflammation by segregating tumor necrosis factor *α* (TNF*α*) and IL-12, and they play a role in tissue destruction [50, 51]. M2 macrophages, or alternatively activated monocytes, generate an enhanced production of anti-inflammatory cytokines, IL-1 receptor antagonist, IL-10, and transforming growth factor (TGF-) beta. Thus, M2 macrophages repair tissues and inhibit inflammation [50, 51]. Furthermore, M2 macrophages express the macrophage mannose receptor, which mediates host defense and the elimination of the substances produced in inflammatory processes [48, 49]. M2 polarization is in fact characterized by an increased expression of innate immunity receptors [18].

The polarization of decidual macrophages toward M2, found in normal pregnancies, indicates that their immunosuppressive activities are critical for maintaining immunological homeostasis during pregnancy [18, 50]. The innate immune response of macrophages is regulated by signaling mediated by pattern recognition receptors, that is, toll-like receptors (TLRs). Recognition of microbes by TLRs on macrophages is the primary host defense mechanism in the decidua [18, 48, 50]. At the end of pregnancy, macrophages with an inflammatory phenotype participate in cervical ripening and in onset of labor [50].

Paradoxically in patients who have undergone IVF, the inflammatory environment generated by performing an endometrial biopsy before implantation entails a higher implantation rate. This is related to a high macrophages/DCs concentration and elevated proinflammatory cytokines [47].

A study of tissue samples from spontaneous abortions and elective terminations of pregnancy has shown an increased population of decidual macrophages in spontaneous abortions. The Fas-ligand (Fas-L) overexpression of these decidual macrophages during spontaneous abortions has also been demonstrated. Fas-L is a transmembrane protein that binds to the Fas receptor and triggers apoptosis to Fas-expressing cells. Therefore, it has been hypothesized that the Fas-L expression by decidual macrophages forms part of M2-like polarization. The Fas-L expression by decidual macrophages could induce apoptosis to Fas-bearing activated T-cells to potentially diminish deleterious maternal immune responses against the semiallogeneic or allogeneic embryo in ED pregnancies [48].

The decidual differentiation of macrophages and DCs is regulated by the granulocyte-macrophage colony-stimulating factor (GM-CSF). In the nonpregnant human endometrium, luminal and glandular epithelial cells are the main source of GM-CSF. A peak in the GM-CSF mRNA levels has been observed during the “window of implantation.” The mRNA for the GM-CSF receptor has been localized in endothelial cells of the spiral artery. GM-CSF is a growth factor for the trophoblast [52]. GM-CSF could play a role in the preeclampsia pathogenesis. GM-CSF levels in decidual cells are higher in patients with preeclampsia if compared with gestational-age matched controls. Besides, the cytokines involved in preeclampsia TNF*α* and IL-1*β* upregulate GM-CSF mRNA in cultured first-trimester human decidual cells [52]. In line with this, GM-CSF levels in blood and GM-CSF/total protein levels in the placenta are significantly higher in gestations with preeclampsia than in normal pregnancies [53]. Therefore, it is feasible to hypothesize that increased GM-CSF in patients with preeclampsia might contribute to DC maturity and the decidual macrophage polarization to M1.

#### 2.1.3. The Complement System

The complement system is an essential component of innate humoral immunity composed of proteins. These mediate the clearance of pathogens, apoptotic cells, and immune complexes by forming a membrane attack complex (MAC), which leads to cell lysis [54, 55]. The complement system can be activated in three pathways: the classic pathway is triggered by antigen-antibody complexes; the alternative pathway is spontaneously and continuously activated; the lectin pathway is triggered by the binding of mannan-binding lectin to mannose residues on the surface of microorganisms [54, 55]. Irrespectively of the mechanism of activation, all the pathways converge to generate C3 convertases, which transform C3 into its active components, C3a and C3b. C3b is a main effector of the complement that tags nonself cells for destruction by phagocytes. C3b also binds to C3 covertases to form C5 convertase, which transforms C5 into C5b and powerful proinflammatory mediator C5a. C5b associates not only with C6, C7, C8, but also with many units of C9, to form a lytic pore that inserts into cell membranes, known as the MAC (C5b-9). C3a and C5a, commonly known as anaphylatoxins given their role in anaphylactic shock, facilitate pathogen clearance by increasing vascular permeability, inducing inflammatory cell chemotaxis, and releasing cytokines. C3a and C5a exert these proinflammatory effects by binding to their respective receptors, C3a receptor (C3aR), and the two receptors for C5, C5a receptor (C5aR; CD88) and C5a receptor-like 2 receptor (C5L2) [55].

Syncytiotrophoblasts, villous cytotrophoblasts, and EVT express the three regulatory proteins of the complement, these being decay-accelerating factor (DAF), membrane cofactor protein (MCP), and CD59 which avoids the formation of the MAC and subsequent cell lysis [55]. Thus, excessive complement activation is prevented in successful human pregnancies thanks to the presence of these three regulatory proteins in trophoblast membranes [55].

The component of complement C1q plays a very important role in trophoblast migration, spiral arteries remodeling, and normal placentation [56]. The decidual endothelial cells (DECs) covering spiral arteries acquire the ability to synthesize C1q. This protein is bound to the cell surface and acts as a physical link between endovascular trophoblasts and DEC to favor the vascular remodeling process [54]. In line with this, C1q-deficient pregnant [C1q (–/–)] rats present the main findings of human preeclampsia: hypertension, albuminuria, endotheliosis, diminished placental VEGF, and elevated levels of soluble VEGF receptor 1 (sFlt-1), with high fetal mortality. Their placentas also display increased oxidative stress and reduced blood flow [56].

Mannose-binding lectin (MBL) activates the lectin pathway of the complement. The level of MBL in the vaginal cavity changes during the menstrual cycle, which is produced locally by vaginal cells [54]. MBL apparently plays a key role in embryonic implantation because an analysis of uterine aspirates obtained upon oocyte capture for IVF has revealed a high level of MBL in patients whose infertility was of unknown etiology as compared to patients who underwent IVF/ICSI for male factor or tubal infertility [54]. Although the serum levels of MBL rise during pregnancy, its function is still to be clarified [54]. Patients with preeclampsia show higher median plasma MBL concentrations when compared to women with uncomplicated pregnancies [57]. Likewise the association of a genetically related MBL polymorphism with MBL diminished the functional activity that protects against preeclampsia [54]. Interestingly, the serum obtained from preeclamptic women has been found to prevent the interaction between EVT and DECs, which avoids the endovascular invasion of trophoblastic cells. Increased serum MBL in women with preeclampsia inhibits the interaction of EVT with C1q, which interferes with the process of EVT adhesion to and migration through DECs [54].

The lectin pathway of complement is also activated by ficolins, which mediate a primitive opsonophagocytosis. They are soluble molecules of the innate immune system that recognize carbohydrate molecules on microbial pathogens, apoptotic, and necrotic cells. Plasma ficolin-2 levels are low in preeclamptic patients if compared with healthy pregnant women. These reduced plasma ficolin-2 concentrations in preeclampsia might contribute to the development of the maternal syndrome of the disease by the impaired removal of the trophoblast-derived material released into the maternal circulation by the hypoxic and oxidatively stressed preeclamptic placenta [58].

Although the complement components are normally high during pregnancy, excessive complement activation, particularly enhanced C5a synthesis, is associated with pregnancy complications such as recurrent abortion, preterm birth, and preeclampsia. C5 can be harmful because it induces antiangiogenic sFlt-1. sFlt-1 sequesters VEGF and PIGF, which are crucial growth factors for normal placental development and for successful pregnancy. Therefore, it has been postulated that C5a can harm angiogenesis by contributing to abnormal placentation, which allows fetal loss in early pregnancy stages or preeclampsia in later stages. Alternatively, C5a can cause preterm birth by inducing cervical ripening and by releasing a large number of birth-prompting mediators [55].

Preeclamptic patients have significantly higher C4d, C3a, and C5b9 levels and substantially lower C3 concentrations than healthy pregnant women [59]. Indeed higher plasmatic levels of C5b9, or excessive terminal complement activation, have been found in preeclamptic patients with intrauterine growth restriction as compared with those presenting normal intrauterine growth [59]. In human pregnancies at between gestation weeks 10 and 15, the plasma levels of activation product Bb (derived from factors B which initiate C3b activation through the alternative pathway initiation complex), activated C3 (C3a), C5-9, and the serum levels of angiogenic factors PiGF, sFLT-1, and sENG, have been quantified. High Bb levels and low PiGF concentrations have been associated with later preeclampsia development [60]. Surprisingly, multiparous women who changed their partner presented higher Bb levels and were 5 times more likely to develop preeclampsia as compared to women who were still with their same partner since their last pregnancy [61].

Women with preeclampsia present significantly higher plasma levels of C5a than women with uncomplicated pregnancy [62]. Before gestation week 20, women who later developed any hypertensive disease related to pregnancy or gestational hypertension showed higher plasma levels of C3a when compared with those who did not develop these diseases [63]. In the placentas of human severe early-onset preeclampsia, a low C3aR expression has been found as compared to women with preterm nonpreeclamptic pregnancies [64].

The activation of complement C3aR through autoantibodies has been revealed to contribute to preeclampsia pathogenesis. The human maternal angiotensin II type 1 receptor agonistic autoantibody stimulates the deposition of complement C3 in placentas and kidneys of pregnant mice through the activation of angiotensin II type 1 receptor. Interference with C3a signaling through a complement C3aR-specific antagonist significantly decreases hypertension and proteinuria in angiotensin II type 1 receptor agonistic autoantibody-injected pregnant mice. Complement C3aR antagonist significantly not only inhibits autoantibody-induced circulating sFlt-1, a well-known antiangiogenic protein related to preeclampsia, but also reduces small placental size with damaged angiogenesis and intrauterine growth restriction. In humans, it has been demonstrated that the placentas of preeclamptic patients present a significantly higher C3 deposition than normotensive controls. In cultured human villous explants, its complement C3aR activation has been seen as an important mechanism that underlies autoantibody-induced sFlt-1 secretion and decreased angiogenesis [65]. The C3aR antagonist may contribute to preeclampsia treatment. Nevertheless, the low C3aR expression in the placentas of women with preeclampsia [64] indicates that further studies are required to evaluate the usefulness of the postulated therapy.

#### 2.1.4. Toll-Like Receptors (TRL)

Cells of the innate immune system respond to infectious microorganisms by pattern recognition receptors, such as TLRs. These recognize the sequences expressed by microbes named pathogen-associated molecular patterns (PAMPs), such as bacterial lipopolysaccharide (LPS) or viral dsRNA. Since both first trimester and term placentas show TLRs, the placenta may recognize pathogens through these receptors and could induce a subsequent immune response [66].

It is known that human first-trimester trophoblasts constitutively secrete chemokines like growth-related oncogene, growth-related oncogene *α*, IL-8, and monocyte chemotactic protein-1 (MCP-1). These chemokines recruit monocytes, NK cells, and neutrophils. The ligation of TLR-3 by viral poly (I:C), or TLR-4 by bacterial LPS, significantly increases the trophoblast secretion of chemokines. This results in elevated monocyte and neutrophil chemotaxis. Moreover, TLR-3 stimulation induces RANTES secretion by trophoblast cells, which is chemotactic for monocytes [66].

A significant increase in the TLR-4 protein expression is observed in placental trophoblasts of preeclamptic patients as compared to normotensive pregnant women [66, 67]. Surprisingly, the expression of TLR-2 and TLR-4 in maternal neutrophils has been found to diminish in preeclampsia when compared with normal pregnant controls of similar gestational ages [68]. Further studies are required to explain the discordant TLR-4 expression between placental trophoblasts and maternal neutrophils in preeclamptic patients.

#### 2.1.5. Decidualized Endometrium Secretion

As a response to the paracrine signals from the trophoblast, the proinflammatory cytokines, chemokines, and angiogenic factors in decidual stromal cells are significantly induced. In line with this, a study was carried out with human endometrial stromal cells decidualized with progesterone, which were treated with either conditional media from human trophoblasts (TCM) or control-conditioned media (CCM) from nondecidualized stromal cells. It revealed that the most overexpressed genes at 12 hours of treatment were chemokines CXCL1 (GRO*α*), IL8, C-X-C chemokine receptor type 4 (CXCR-4), and other genes implicated in the immune response, such as pentraxin 3 (PTX3), IL6, and TNF*α*-induced protein 6 (TNFAIP6), or metaloproteinases (MMP1, MMP10, and MMP14). The downregulated genes were growth factors, such as IGF1, FGF1, and the genes involved in Wnt signaling. Paracrine interactions between EVT and the maternal decidua are therefore essential for successful embryonic implantation, which occurs in an enriched cytokine/chemokine environment where stromal cells' mitotic activity is limited in the invasive implantation phase [69]. A prior* in vitro* study also revealed that the most overexpressed genes by endometrial stromal cells during implantation, due to the effect of the trophoblast, were those involved in inflammatory response, immune response and chemotaxis (PTX3, IL8, IL1 receptors, IL18 receptor, and TNFAIP6), cell growth regulators (IGF-binding proteins 1 and 2), and signal transduction. Downregulated genes were those involved in proteolysis (MMP11) and cell death, transcription factors, and the genes involved in the humoral immune response (CD24 antigen) [70].

The main secretory product of a pregnant woman's decidualized endometrium is insulin-like growth factor binding protein-1 (IGFBP1). Its interaction with the *α*5*β*1 integrin of the EVT cell surface triggers its migration in an IGF-independent manner. Whereas decidual IGFBP1 production increases progressively during the first and second trimesters of uncomplicated pregnancies, women destined to develop preeclampsia present low serum levels of IGFBP1, which may indicate decidual dysfunction [71].

### 2.2. Antigens and Trophoblast Activity

#### 2.2.1. The Trophoblast HLA Expression Pattern

The vast majority of the trophoblast that comes into contact with maternal tissue does not possess the antigenic determinants required for maternal T-cell activation; indeed it prevents the potential maternal antifetal rejection. The syncytiotrophoblast that is the main trophoblast to come into contact with the maternal immune system lacks classic class I and II HLA antigens. EVT has an invasive phenotype and forms columns of cells that invade the maternal decidua and replace the endothelium of spiral arteries. This EVT expresses a single class I HLA expression pattern with nonpolymorphic molecules, which include HLA-E, -F, -G, and -C [29].

HLA-G is crucial in maternal tolerance to the semiallogeneic or allogeneic fetus in ED pregnancies. The HLA-G expression in EVT has been found throughout pregnancy [26]. With Fas-L, soluble HLA-G induces CD8+ T cell apoptosis [24, 29]. HLA-G expressed in EVT inhibits not only cytotoxic T lymphocyte responses, but also NK cell functions. A leader peptide of HLA-G forms a complex with HLA-E on the trophoblast cell surface and binds to the CD94/NKG2 receptor in NK cells. NK cell activity is subsequently inhibited [24].

HLA-F is also found in EVT. Although poorly expressed during the first trimester, its expression increases during pregnancy [27]. HLA-E, which is found in all the cells expressing HLA-C or HLA-F, is localized mainly on the EVT surface that invades the maternal decidua. Like HLA-F, it can promote fetal growth as its expression coincides with the rapid fetal growth period [27]. Although polymorphic HLA-C is also present in EVT, it is not as highly polymorphic as HLA-A and HLA-B. Of all the HLA class I molecules expressed by EVT, only HLA-C displays the variability required to constitute a fetal alloantigen, and it is recognized by maternal uNK cells through their KIR receptors [29].

#### 2.2.2. Fas Ligand

Fas-L is a transmembrane protein [72]. Fas-L from fetal EVT or maternal decidual cells, coupled with soluble HLA-G from EVT, induces CD8+ T-cell apoptosis [23, 28], thus increasing maternofetal immune tolerance. Isolated first-trimester trophoblast cells have been described to not show FasL on their membrane but to also express a cytoplasmic form. This intracellular FasL is constitutively secreted by trophoblast cells through the release of microvesicles. After the disruption of these microvesicles, the secreted FasL induces T-cell apoptosis through the activation of the Fas pathway [72]. This knowledge has been supported by a subsequent* in vivo* study, which has only found FasL production and storage in first-trimester human syncytiotrophoblast, but not in the cytotrophoblast [73]. On the other hand, it has been recently reported that the Fas-L A-670G polymorphism is associated with increased risk of preeclampsia [74]. Therefore, the desire to gain a better understanding of the fetal FasL expression and its contribution to maternofetal tolerance may inspire further studies.

#### 2.2.3. Indoleamine 2,3-Dioxygenase

Indoleamine 2,3-dioxygenase (IDO) is an enzyme that degrades the tryptophan amino acid. It is expressed in both EVT and villous trophoblasts in humans, where it may inhibit maternal T-cell activation through the deprivation of tryptophan T-cells [24]. The serum tryptophan levels decrease from the first trimester of human pregnancy [24]. The pharmacological inhibition of IDO activity in murine pregnancy has been demonstrated to induce maternal T-cell-mediated rejection of the allogenic, but not the syngenic concept. Nevertheless, the genetic deletion of IDO in mice results in normal litter size as compared to IDO-sufficient control mice. It is worth noting that lack of IDO in mammals can be compensated by the tryptophan dioxygenase enzyme, which induces tryptophan catabolism [24].

#### 2.2.4. The B7 Family

The B7 family molecules are transmembrane proteins that belong to the immunoglobulin superfamily [75]. Optimal maternal T-cell activation requires the connection between the T-cell receptor (TCR) and the HLA antigenic peptide of the antigen-presenting cell (APC). In order to provoke efficient T-cell activation, a positive costimulatory signal is required, which is mediated by the interaction between CD28, which is constitutively expressed in most mature T-cells, and molecules B7-1 and B7-2 exposed by APC [74]. Interestingly, blockade of B7-1 and B7-2 at the time of murine implantation has been reported to induce the inhibition of maternal fetus rejection in abortion-prone CBA/JxDBA/2 matings [76]. Moreover, the frequency of B7-1 and B7-2 expressing activated monocytes in peripheral blood of preeclamptic patients is lower than in normal pregnant woman [77].

B7-1 and B7-2 also bind in another receptor in T-cells, cytotoxic lymphocyte antigen-4 (CTLA-4). Their union provides an inhibitory signal that plays a key role in the negative regulation of the immune system [75]. Fetal tissues express CTLA-4 at the maternofetal interface during pregnancy. Susceptibility to recurrent spontaneous abortion mediated by a polymorphism in the CTLA4 gene has been suggested [24].

Another costimulatory pathway that plays a role in peripheral tolerance is defined by the programmed death-1 receptor and its ligands, PDL1 and PDL2. In pregnancy, while PDL1 or B7-H1 is expressed by all the trophoblast populations, PDL2 or B7-DC is present in the syncytiotrophoblast in early pregnancy [75]. It is known that PDL1 is essential to maintain maternofetal tolerance, and its blockade or deficiency results in poor fetal survival and a shift toward Th1 placental cytokines [24].

### 2.3. Systemic Maternal Immunological Response during Pregnancy

#### 2.3.1. Characteristic Cytokine Profile of T Helper 2 Cells

Th lymphocytes can be classified as Th1 or Th2. Initially, it was suggested that the human fetus is not rejected by the maternal immune system due to the prevalent cytokine production of Th2 cells. The Th2 cytokines produced at the maternal-fetal interface would inhibit Th1 responses, leading to fetal survival [78]. Yet while Th2 cells predominate in early pregnancy decidua, Th1 cells prevail in the nonpregnant endometrium, particularly in the proliferative phase. Thus, the Th1/Th2 ratio peaks in the proliferative endometrium and significantly decreases in the secretory phase and reaches its lowest level in the early pregnancy decidua [79]. Similarly, the Th2 cytokine expression, specifically IL-6 and IL-10, is 10-fold higher in the early pregnancy decidua as compared to the nonpregnant endometrium [80]. Besides, progesterone stimulates a Th2-type response, decreases inflammatory cytokines, and restrains allogeneic responses to allow fetal survival [18].

The characteristic cytokines of Th2 cells are IL-4, IL-5, IL-6, IL-9, IL-10, and IL-13. Although these cells participate in the development of humoral immunity against extracellular pathogens, they also repress the functions of phagocytic cells. Th1 cells not only synthesize interferon-g (IFN-g), IL-2, and tumor necrosis factor-a (TNF-*α*), but also trigger cell-mediated immunity and phagocyte-dependent inflammation [81].

A tendency for immune Th1 responses has been found in human pregnancy-related complications, such as recurrent spontaneous abortions. Significantly higher serum levels of Th2 cytokines, IL-6, and IL-10 and considerably lower levels of the Th1 cytokine, IFN-g, have been reported in normal pregnancy as compared to unexplained recurrent pregnancy losses [82]. Accordingly, patients reporting recurrent pregnancy losses and infertile women with multiple implantation failures after IVF present increased T helper 1 cytokine responses by circulating T-cells [81]. The injection of each Th1 cytokine, like IFN-g, TNF-*α*, and IL-2, or the coadministration of these, in pregnant mice significantly increased fetal resorption [81].

However, the function of a major chemokine in the Th1 response, RANTES, may prove essential for modulating the responses of specific T-cells for alloantigens during normal pregnancy. Indeed, successful pregnancies are accompanied by increased serum levels of RANTES, which are lower in patients who suffer recurrent abortions. It has been demonstrated* in vitro* that RANTES specifically suppresses alloactivated maternal T-cells. So the high levels of progesterone present during normal pregnancy, particularly on the maternofetal surface, can be predictors of RANTES production at levels required to induce a tolerogenic immune response locally [18].

RANTES (also known as CCL5) is a proinflammatory chemokine that can act as a modulator of alloantigen-specific T-cell responses in healthy pregnancy [18]. Whereas in successful pregnancies the serum levels of RANTES are high, they are low in recurrent spontaneous abortions [83]. Indeed, RANTES accurately suppresses alloactivated maternal T-cells [84]. Thus, RANTES might cooperate in the maternal tolerogenic immune response to allow trophoblast cell survival and migration [18].

Th2 preponderance in normal pregnancy shifts to Th1 predominance in preeclampsia. It is known that in peripheral blood in preeclampsia, the percentage of Th1 cells and the Th1/Th2 ratio are significantly higher, while the percentage of Th2 cells is significantly lower than in the third trimester of healthy pregnancy [85]. A change to Th1-type immunity is expressed in the serum of preeclamptic patients by an increase in the IL2/IL4 and IFNg/IL4 ratios. In addition, preeclampsia is associated with a proinflammatory systemic environment due to the elevated circulating levels of proinflammatory cytokines IL-6 and TNF-alpha, chemokines IL-8, IP-10, and MCP-1, and adhesion molecules intercellular adhesion molecule 1 (ICAM-1) and vascular cell adhesion protein 1 (VCAM-1) as compared to normal pregnancy. Surprisingly, the increased IP-10, MCP-1, ICAM-1, and VCAM-1 concentrations in preeclamptic patients correlate significantly with blood pressure values and liver and renal function parameters [85]. In line with this, the peripheral blood mononuclear cell production of IL-12, which induces Th1 responses, diminishes in normal pregnant women but increases in preeclamptic patients [86].

#### 2.3.2. Immunosuppressor Activity of T Regulatory Cells

In the maternal immune response against the fetus, which may be considered a semiallograft or an allograft in pregnancies by ED, the role of CD4+CD25+Foxp3+ Treg cells is particularly relevant. The transcriptional regulatory protein forkhead box P3 (FOXP3) is a transcriptional repressor required for the development and function of Treg cells [87]. It has been identified in deciduas CD4+ T-cells expressing FOXP3 with high levels of CD25 (CD4+CD25^bright^FOXP3+) or low levels of CD25 (CD4+CD25^dim⁡^) [88]. Whereas decidual CD4+CD25^bright^ Treg cells are involved in the regulation of immune responses in humans, decidual CD4+CD25^dim⁡^ T-cells display an activated phenotype by expressing raised levels of CD69 and low levels of FOXP3 and cytotoxic T lymphocyte antigen (CTLA)-4 [88]. Decidual CD4+CD25^bright^ Treg cells contribute to the maternal immune tolerance of fetal antigens, since deciduas in early human pregnancy contain abundant CD4+CD25^bright^ Treg cells that express CTLA-4 at high levels. These cells prevent the proliferation of autologous CD4+CD25– T-cells. Moreover, the proportion of decidual CD4+CD25^bright^ T-cells is substantially lower in spontaneous abortion as compared to induced abortions [89].

Treg cells are essential in the induction and maintenance of MHC class II antigen-specific tolerance. Although HLA II is not expressed in villous or EVT, the trophoblastic cell debris containing the intracellular fetal HLA-DR antigen circulates in maternal blood. It has been postulated that immature DCs, acting as APCs, catch these debris and induce peripheral tolerance through the induction of Treg cells. The immune regulation of CD4+ T-cells is carried out mainly by Treg cells. The apoptosis of CD8+ T-cells is induced by the soluble HLA-G and Fas ligand expressed in EVT. The regulation of both CD4+ and CD8+ T-cells results in maternofetal tolerance [29].

Treg cells also enhance the maternal tolerance of the fetus through the expression of CTLA-4 on their surface. The ligation of CTLA-4 by transmembrane protein B7 of APCs results in an increased IDO expression on decidual and peripheral blood DCs and monocytes/macrophages [90]. IDO restrains the availability of tryptophan to T-cells [18].

Circulating Treg cells increase during early pregnancy, reach a higher level during the second trimester, and decline postpartum [91]. Estrogen has been suggested to promote maternofetal tolerance by increasing Treg cells since the treatment of naïve mice with E2 increases both the CD25+ cell number and the FoxP3 expression level. In addition, estrogen treatment and pregnancy induce a similar FoxP3 protein expression [87]. In line with this,* in vivo* and* in vitro* elegant mice models have provided evidence that progesterone increases the proportion of CD4+CD25+ Treg cells and IL-10 expression and enhances their suppressive function. Additionally at equivalent physiological doses to midterm pregnancy, progesterone, but not estradiol, converts TCD4+CD25− T-cells into CD4+CD25+ Treg cells. It has therefore been suggested that progesterone extends Treg cell populations by means of nuclear progesterone receptors. Besides, RU 486 significantly decreases the amount and function of Treg cells at the maternofetal interface before the onset of induced abortion. The significantly reduced Foxp3 expression has been reported to be accompanied by a significant increase in proinflammatory factors [92].

A subpopulation of TCD4+ effector cells, Th17, differs from Th1, Th2, and Treg cells. Th17 cells secrete IL-17 and express CC chemokine receptor type 6 (CCR6) [93]. Whereas the prevalence of Tregs lowered, that of Th17 cells increased in both the peripheral blood and decidua of patients with unexplained recurrent miscarriage as compared to healthy early pregnant women [94]. Interestingly, the IL-17 expression can be inhibited by Treg. Patients with unexplained recurrent miscarriage display diminished suppressive activity of Tregs in Th17 cells when compared with healthy women who underwent early elective abortion [93].

Nowadays, it is believed that unexplained recurrent spontaneous abortions could be an alloimmune disease associated with defective maternofetal tolerance in which Treg cells play a key role. As Foxp3 is a crucial regulatory factor for the development and function of Treg cells; Foxp3 gene deficiency suppresses the regulatory function of Treg cells. Accordingly, a significant association has been found between Foxp3 gene polymorphisms rs3761548A/C and rs2232365A/G and unexplained recurrent abortions in a Chinese female population [95].

Tregs are essential for pregnancy maintenance, and low levels have been found in pregnancy complications. Thus not only women with unexplained recurrent spontaneous abortions, but also patients with preeclampsia display low levels of Tregs in both maternal blood and placenta. In fact in preeclamptic patients, the percentage of CD25^bright^ cells in the CD4+ T cell population in peripheral blood mononuclear cells is considerably lower than in women with normal pregnancies and nonpregnant healthy controls. Moreover, placental samples from preeclamptic patients show a low percentage of FoxP3+ cells in CD3+ T-cells as compared to those reported in normal pregnancy subjects. It has been suggested that cytotoxic T-cells increase at the decidua basalis in preeclampsia since the CD8+ T/CD3+ T-cells ratio in placental preeclamptic samples was much higher than in the samples taken from healthy pregnancies [96]. The frequency of conventional CD4+ CD25high FoxP3+ Tregs and that of nonconventional CD4+ CD25– FoxP3+ Tregs diminish in peripheral blood in preeclamptic patients as compared to healthy pregnant women [97]. In addition, the prevalence of Th17 cells and the Th17/Treg ratio increases in peripheral blood in preeclampsia as compared with normal pregnancy [98].

Since the complete fetal genome is allogeneic to the mother in ED pregnancies, maternofetal immune tolerance is particularly essential for pregnancy success. The substantially large number of T CD4+ and NK cells in the basal plaque of placentas from ED pregnancies, if compared with those from nondonor IVF pregnancies, may reveal that maternal immune tolerance against the fetal allograft is enhanced [99].

Strangely enough, it has been recently suggested in maternal tolerance to the semi- or allogeneic fetus in ED pregnancies that peripheral or extrathymic Treg cells are vital as they block the immune response to foreign antigens. Conversely, thymic Treg cells suppress autoimmunity [100, 101].

*Semen Exposure for the Induction of T Regulatory Cells.* Besides exposure to trophoblastic cell debris, exposure to sperm may also induce HLA class II-specific tolerance. HLA-DR antigens expressed on sperm might induce HLA II antigen-specific tolerance. Treg cells play a central role in inducing and maintaining this process [29]. Murine models have shown that Treg cells are activated by male antigens [102]. As a matter of fact, seminal fluid expands the pool of Treg cells in the para-aortic lymph nodes draining the uterus [103] and induces the accumulation of Treg cells in the uterus prior to embryo implantation [104]. Indeed Treg cells are essential in embryo implantation. Treg cells accumulate in the mouse uterus in the receptive phase of the estrus cycle, and seminal fluid further promotes Treg expansion [105]. On the other hand, soluble HLA class I in seminal fluid may induce HLA I-specific tolerance. NK cells play a key role in this tolerance induction. Such exposure may increase maternal immune tolerance to paternal HLA class I and II antigens before pregnancy [29].

In pregnancies achieved by donated spermatozoa, women have not been previously exposed to semen and the fetus is a semiallograft to the mother. Since the risk of preeclampsia in donated spermatozoa is very high (18.2%), semen exposure would reduce the risk of preeclampsia [29]. Along these lines, risk of preeclampsia has been studied with intracytoplasmatic sperm injection (ICSI) using either ejaculated sperm or surgically obtained sperm, and both cases involve exposure to seminal fluid. Whereas in ICSI with ejaculated sperm sperm exposure exists, exposure is absent in ICSI with surgically obtained sperm. The risk of preeclampsia in ICSI with ejaculated sperm is the same as that for IVF cases, 4%. Oddly enough, the risk of preeclampsia in ICSI using surgically obtained sperm is 11%, which is significantly higher than in ICSI with ejaculated sperm [106]. In addition, as the risk of preeclampsia in ED pregnancies with former exposure to the partner's semen is high (16.00%), the allogeneic fetus may constitute a risk factor of preeclampsia. In donated embryo transfer cases, the fetus is allogeneic to the mother, and no former semen exposure is involved. In such cases, risk of preeclampsia is extremely high (33.00%) [29]. These findings highlight the importance of sperm exposure in inducing maternofetal immune tolerance.

Onset of preeclampsia may be related to the gradual decrease of Treg cells, which induce paternal antigen-specific tolerance during the third trimester of pregnancy [29, 91]. In addition, a protective effect of multiparity in preeclampsia has been described. Despite the possibility of memory T-cells decreasing after delivery, seminal priming may maintain their number at a certain level. Thus in a second pregnancy with the same partner, the number of memory T-cells may rapidly increase. This protective multiparity effect in preeclampsia would be lost with a change of partner [29]. It is also noteworthy that the longer the interval between second and third deliveries with the same partner, the higher the risk of preeclampsia. This finding may be explained by the progressive decrease in memory T-cells after delivery in the second or third pregnancy. Memory T-cell levels reach their lowest levels at more than 10 years after the last delivery, and seminal priming maintains these tolerance-inducing T-cells at a low level. Therefore, in a subsequent pregnancy, some of these women may not achieve adequate tolerance which, in turn, raises the risk of preeclampsia [29].

#### 2.3.3. B Lymphocytes and Maternal Antibodies against Fetal HLA

Regarding the adaptive maternal humoral immune response during pregnancy, paternal anti-HLA antibodies have been observed in multiparous mouse animal models. Similarly, fetal HLA-specific B-cells have been detected in murine pregnancies [107]. B-cells are capable of producing antibodies [108].

In human pregnancies, fetal antigens induce an adaptive maternal humoral immune system response. Accordingly, maternal antibodies against fetal HLA can be generated, which are especially prone to increase when the HLA mismatches between the mother and fetus are high. Since ED pregnancies may be associated with a larger number of HLA mismatches than spontaneously conceived pregnancies, women with ED pregnancies might produce higher levels of antibodies. It remains unknown whether adverse clinical consequences occur as a result of the maternal humoral immune response to fetal antigens. In fact, antipaternal HLA antibodies and antifetal T-cells are present in many normal pregnancies [107].

In preeclamptic patients, the autoantibody against angiotensin II type I receptor (AT1-AA) has been found. It binds to the AT1 receptor, which is highly expressed in the placenta and triggers the activation of an intracellular cascade, which results in the production of antiangiogenic factors sFlt1 and endoglin [108]. B-cells form part of the adaptive maternal cellular immune response. Two B-cell subpopulations are B1 and B2 cells. Whereas B1 cells develop during fetal and perinatal life, B2 cells are produced during postnatal life. B1 cells may be subdivided into B1a and B1b cells based on the expression of cellular marker CD5 by B1a cells, but not by B1b cells. B2 and B1b cells produce adaptative antibodies upon antigen stimulation, while B1a cells synthesize natural antibodies in the absence of antigenic stimuli [108]. It is known that AAT1-AA autoantibodies are produced by the CD19+CD5+B1a cells, but not by CD19+CD5–B2 cells, obtained from peripheral blood of nonpregnant women and stimulated* in vitro* with serum from preeclamptic women [109]. During human pregnancies, proportions of CD5^+^ B1a cells significantly decrease. Thus, it has been suggested that the reduction of circulating B1a cells during pregnancy may contribute to maternofetal immune tolerance since these cells are the main producers of poly-reactive antibodies [110]. In pregnant women with uncomplicated pregnancies, CD19+CD5+ levels are significantly lower toward the third trimester, while CD19+CD5+ levels remain high in preeclamptic patients [109].

## 3. Maternal Tolerance in Pregnancy by Egg Donation

In ED pregnancies, the fetus is allogeneic to the mother. Fetal HLA arises from the donor's ovule and from the biological father of the future newborn child. In spontaneous pregnancies, the fetus is semi allogeneic to the mother. It has been shown that hyperactivation of Th1 and Th2 by an allogeneic fetus is specific for ED pregnancy in the first trimester of pregnancy if compared with IVF pregnancies and pregnancies by natural conception. Another regulatory counteractive mechanism in ED pregnancies is reflected by the preferable activation of Th2 and the relative suppression of the Th1 chemokine expression [111].

The larger number of mismatches in the five most immunogenic HLA antigens (HLA-A, -B, -C, -DR, and -DQ) in ED pregnancies may have clinical consequences. Indeed, the healthy uncomplicated term pregnancies containing a HLA-C mismatched child induce a higher percentage of CD4+CD25^dim⁡^ activated-T cells in decidua parietalis and contain functional CD4+CD25^bright^ regulatory T-cells in decidual tissue when compared with HLA-C-matched pregnancies [111]. Moreover, a significant correlation between the total number of HLA-A, HLA-B, HLA-C, HLA-DR, and HLA-DQ mismatches and the percentage of activated CD4+CD25^dim⁡^ T-cells in decidua parietalis has been described. Therefore, further activation by fetal HLA-A, HLA-B, HLA-DR, and HLA-DQ may occur in pregnancy. As trophoblast cells do not express these HLA molecules, the microchimeric fetal cells that express HLA antigens before entering the decidual tissue may activate a number of decidual T-cells in the periphery. Activation in the decidua might occur by HLA-C, which explains the prevailing effect of an HLA-C match on the functional faculties of decidual T-cells [111].

A meta-analysis revealed that the OR for pregnancy-induced hypertension after ED, as compared to conventional assisted reproductive techniques, was 2.57 (95%CI, 1.91–3.47). Moreover, the OR for pregnancy-induced hypertension after ED, if compared to the control naturally conceived pregnancy group, was 6.60 (95%CI, 4.55–9.57) [112]. A subsequent retrospective study reported that the incidence of both gestational hypertension and preeclampsia was significantly higher in ED pregnancies than in pregnancies by autologous IVF (24.7% versus 7.4%, and 16.9% versus 4.9%, resp.,) [113].

Although the literature describes higher maternal morbility in ED pregnancies (pregnancy-induced hypertension, preeclampsia, bleeding complications during the first trimester), a higher rate of complications (intrauterine growth restriction, congenital anomalies) for the fetus or newborn has not been demonstrated [4]. Nonetheless, ED pregnancies are more likely to end in preterm birth than pregnancies by autologous IVF (34% versus 19%) [98]. It is known in spontaneous preterm births that maternal anti-HLA class I seropositivity is significantly higher than in term births [114]. ED pregnancies (fetal allograft) may be associated with higher maternal anti-HLA I seropositivity than pregnancies by autologous IVF or those spontaneously conceived (fetal semi-allograft). Therefore, the higher levels of maternal anti-fetal HLA I antibodies in ED pregnancies may be the cause of the higher incidence of preterm birth in these pregnancies when compared with autologous IVF or spontaneous pregnancies. Typification of donors' and recipients' HLA to select haploidentical combinations can be considered in ED pregnancies in order to make them more immunologically comparable to spontaneous pregnancies.

## 4. Conclusion

During pregnancy, the maternal immunological response allows maternal tolerance to the semiallogeneic or allogeneic fetus in ED pregnancies. A defective maternofetal immune response may contribute to the development of pregnancy-related complications, such as bleeding complications during the first trimester, pregnancy-induced hypertension, preeclampsia, or preterm birth. Therefore, suitable knowledge of the maternal immune response during pregnancy will enable us to understand the etiopathogeny to elucidate prevention and to improve the treatment of these pathologies.
